# The Interplay between HIV-1 Gag Binding to the Plasma Membrane and Env Incorporation

**DOI:** 10.3390/v12050548

**Published:** 2020-05-16

**Authors:** R. Elliot Murphy, Jamil S. Saad

**Affiliations:** Department of Microbiology, University of Alabama at Birmingham, Birmingham, AL 35294, USA; murph742@uab.edu

**Keywords:** retroviruses, HIV-1, Gag, matrix, envelope, membrane, cytoplasmic tail, nuclear magnetic resonance (NMR)

## Abstract

Advancement in drug therapies and patient care have drastically improved the mortality rates of HIV-1 infected individuals. Many of these therapies were developed or improved upon by using structure-based techniques, which underscore the importance of understanding essential mechanisms in the replication cycle of HIV-1 at the structural level. One such process which remains poorly understood is the incorporation of the envelope glycoprotein (Env) into budding virus particles. Assembly of HIV particles is initiated by targeting of the Gag polyproteins to the inner leaflet of the plasma membrane (PM), a process mediated by the N-terminally myristoylated matrix (MA) domain and phosphatidylinositol 4,5-bisphosphate (PI(4,5)P_2_). There is strong evidence that formation of the Gag lattice on the PM is a prerequisite for the incorporation of Env into budding particles. It is also suggested that Env incorporation is mediated by an interaction between its cytoplasmic tail (gp41CT) and the MA domain of Gag. In this review, we highlight the latest developments and current efforts to understand the interplay between gp41CT, MA, and the membrane during assembly. Elucidation of the molecular determinants of Gag–Env–membrane interactions may help in the development of new antiviral therapeutic agents that inhibit particle assembly, Env incorporation and ultimately virus production.

## 1. Introduction

Human immunodeficiency virus type 1 (HIV-1), the causative agent of acquired immune deficiency syndrome (AIDS), is responsible for one of the deadliest viral pandemics in human history. Even though advances in treatment, particularly in antiretroviral therapies (ARTs) and patient care, have drastically reduced the morbidity and mortality of infected individuals worldwide [1,2], there are still ~36 million adults and 1.7 million children (<15 years) living with HIV-1 today (UNAIDS report, 2019). While ARTs have been effective in combating the AIDS epidemic, they are not capable of eradicating the virus from the body entirely, requiring patients to undergo indefinite treatments [3] which can lead to adverse effects including drug toxicity and drug resistant mutations in the viral genome ([4,5,6] and references therein). Because HIV-1 still represents a major risk to human health and safety around the globe, it is vital that efforts continue to focus on new targets within the virus replication cycle. Three-dimensional molecular structures often provide detailed information on biological mechanisms, which significantly aid in the development of therapeutic interventions [7]. The development of new drugs designed to disrupt the viral assembly process could enhance the effectiveness of modern treatment options or overcome issues of drug resistance and toxicity. This review will focus on illustrating the structure and function of essential elements involved in assembly and incorporation of the envelope (Env) protein into budding particles which are indispensable steps in virus replication. In particular, we will focus on the structures and functions of the matrix (MA) domain of the Gag polyprotein and the cytoplasmic domain of the gp41 subunit of Env (gp41CT), both of which are implicated in these processes. Detailed molecular characterization of this key step may aid in the development of new therapeutic strategies that inhibit virus assembly and Env incorporation.

## 2. Gag Assembly on the Plasma Membrane

**Figure 1:**
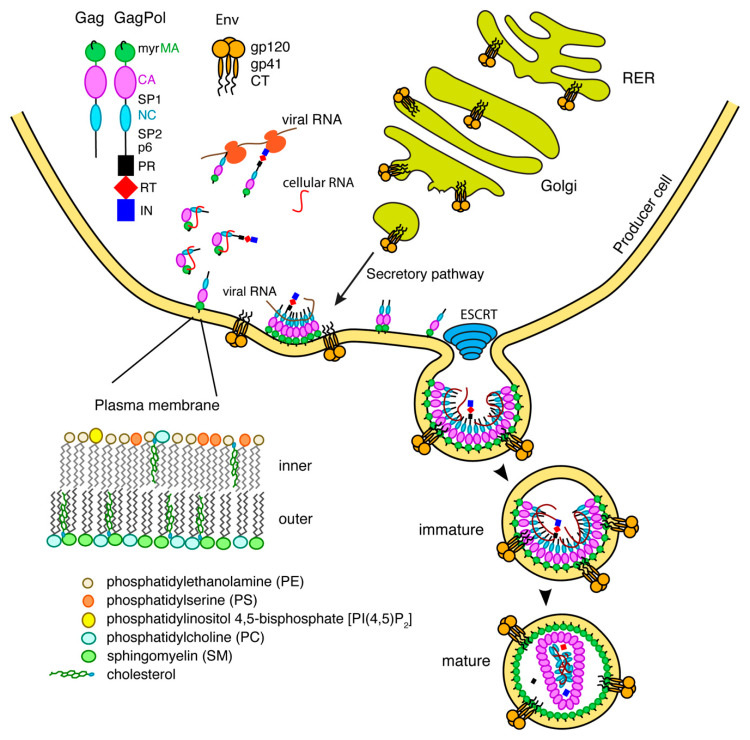
Late phase of the HIV-1 replication cycle. During the late phase of the HIV-1 replication cycle, the newly synthesized Gag and GagPol precursor polyproteins, containing MA, CA, NC, p6 and spacer peptides [and protease (PR), reverse transcriptase (RT), and integrase (IN) for GagPol], are targeted to the PM for assembly. Gag and GagPol polyproteins are anchored to the plasma membrane via insertion of the amino-terminal myr group into the lipid bilayer and by specific interactions with PI(4,5)P_2_. In the meantime, through an independent pathway, the Env glycoprotein which is synthesized in the rough endoplasmic reticulum (RER), traffics to the PM via the secretory pathway to the Golgi. Env is then incorporated into the budding particle in a process mediated by the endosomal sorting complex required for transport (ESCRT). The replication cycle is concluded by the maturation step, which involves cleavage of Gag into individual domains and subsequent formation of the CA core.

**Figure 2:**
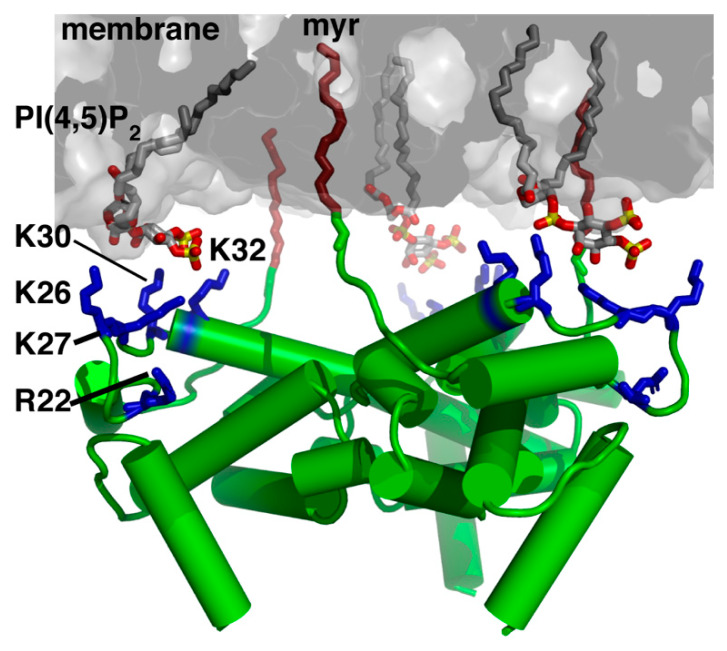
A model of the HIV-1 MA trimer bound to membrane. MA–membrane interaction is mediated by the myr group, basic residues (Arg^22^, Lys^26^, Lys^27^, Lys^30^ and Lys^32^) in the high basic region (HBR; blue), and the polar head of PI(4,5)P_2_. Membrane bilayer was generated in VMD membrane builder plug-in [53]. PI(4,5)P_2_ was generated in Avogadro [54]. The myr-exposed MA trimer was constructed by superimposition with the x-ray structure of myr(–)MA (PDB ID: 1HIW). Note that the N-terminal Met, which is absent in the myristoylated protein, is designated as residue 1. In contrast, other studies considered the N-terminal Gly of the myristoylated protein as residue 1.

The 55 kDa Gag polyprotein precursor is composed of four functionally distinct domains known as MA, capsid (CA), nucleocapsid (NC) and p6, as well as two spacer peptides (SP1 and SP2). GagPol, which contains the elements of Gag as well as the viral enzymes protease (PR), reverse transcriptase (RT) and integrase (IN), is produced and incorporated at lower levels relative to Gag (~5%) due to an infrequent ribosomal frameshift event [8,9]. Subsequent to their synthesis, Gag polyproteins are targeted to the assembly sites on the inner leaflet of the plasma membrane (PM) for particle budding and release (Figure 1) [10,11,12,13,14,15,16]. Gag binding to the PM is mediated by MA, which for most retroviruses, including HIV-1, contains an N-terminal myristoyl group (myr) and a highly basic region (HBR; Figure 2) ([17,18] and references therein). NC, the RNA binding domain of Gag, engages with specific motifs on the unspliced dimeric viral RNA in order to transport the genetic material to sites of assembly for packaging into virus particles [19,20]. Coalescences of Gag, GagPol and viral RNA at assembly sites nucleate the formation of a Gag lattice, driven by lateral intermolecular contacts within the CA and SP1 domains (Figure 1) [18,21,22]. Studies have established that Gag association with membranes is regulated by electrostatic and hydrophobic interactions, protein multimerization, cellular and viral RNA and recognition of specific phospholipids as well as sensitivity to lipid acyl chains [16,23,24,25,26,27,28]. 

Cryo-electron tomography data revealed that the Gag lattice manifests as a cage of interconnected hexamers that cover most of the inner surface of the viral envelope [29,30]. As the lattice grows, the associated membrane curves outward, eventually leading to the formation of an immature virus particle with only a narrow stretch of membrane connecting the viral envelope to the cellular PM. The immature lattice forms a spherical shape in which the Gag proteins are arranged radially with MA on one end and p6 on the other end pointing toward the center (Figure 1) [31]. The p6 domain recruits the cellular endosomal sorting complex required for transport (ESCRT), which catalyzes the scission of the particle from the membrane [32,33]. Virus maturation occurs when the virally encoded protease cleaves the Gag and GagPol precursors into their constitutive proteins [34]. Approximately 1200–2000 copies of the mature CA protein undergo conformational changes and condense to form the conical core of the virus, which surrounds the NC–RNA complex [18,31]. This core takes the shape of a fullerene cone, which consists of ~250 CA hexamers and 12 pentamers [31].

## 3. Structure and Function of MA

Solution NMR spectroscopy and x-ray crystallography studies revealed that the HIV-1 MA protein is predominantly globular and composed of five α-helical domains and a short 3_10_ helix (Figure 2) [35,36,37,38,39,40,41,42]. The C-terminal helix of MA extends away from the globular region, progressively transitioning into a random coil which serves as the linker between the MA and CA domains in the immature Gag protein [40]. The HBR in MA is highly conserved in almost all retroviral MA proteins and serves as the binding site for the polar head of acidic phospholipids in the inner leaflet of the PM [17,18]. Myristoylation of HIV-1 MA is essential for efficient Gag binding to membranes [43,44,45,46,47]. The finding that HIV-1 Gag binds to membranes more efficiently than the isolated MA protein led to the hypothesis that the myr group is exposed in Gag and sequestered in the MA protein, which has become known as “the myr switch mechanism” [44,45,48,49,50]. NMR and analytical ultracentrifugation studies revealed that the myr group can adopt sequestered and exposed conformations in the MA protein, that the MA protein resides in monomer–trimer equilibrium and that myr exposure is coupled with protein trimerization [41]. Exposure of the myr group can also be modulated or triggered by other factors including the solution pH, inclusion of the CA domain and binding of calmodulin (CaM) [41,51,52].

Earlier genetic studies have shown that mutations in the HBR in HIV-1 MA led to Gag targeting to the cytoplasm and/or to intracellular compartments [55,56,57]. These results were later explained by the discovery that Gag localization on the PM is highly dependent on a specific interaction between the HBR and phosphatidylinositol 4,5-bisphosphate (PI(4,5)P_2_) [15,16,24,58], a minor PM lipid that fulfills many cellular functions by acting as a substrate for numerous proteins [59,60,61,62,63]. Phosphoinositides, which are derived from phosphatidylinositol and comprise a diacylglycerol moiety linked to a D-myo-inositol ring via a phosphodiester linkage, can be monophosphorylated (PI(3)P, PI(4)P and PI(5)P), bisphosphorylated (PI(3,4)P_2_), PI(3,5)P_2_ and PI(4,5)P_2_) and trisphosphorylated (PI(3,4,5)P_3_). Phosphoinositides can be interconverted by a series of kinases and phosphatases which serve to regulate their distribution throughout the cell. This establishes a signaling mechanism by which various cytoplasmic proteins that preferentially bind to phosphoinositides are targeted to specific membrane domains within the cell [61,62,63,64,65]. PI(4,5)P_2_ and PI(3,4,5)P_3_ are predominantly localized at the inner leaflet of the PM [59,60,61,62,63]. Numerous cellular proteins, such as those containing the pleckstrin homology domains, are often directed to the PM through specific interactions with PI(4,5)P_2_ or PI(3,4,5)P_3_ [66,67,68,69]. 

Over-expression of phosphoinositide 5-phosphatase IV (5ptaseIV), which reduces PI(4,5)P_2_ levels by hydrolyzing the phosphate at the D5 position of PI(4,5)P_2_, resulted in a significant reduction in HIV-1 Gag’s PM localization and attenuation of virus production [16]. PI(4,5)P_2_-dependent Gag assembly and/or PM localization have been observed for other retroviruses such as HIV-2, Mason-Pfizer monkey virus (MPMV), murine leukemia virus (MLV), feline immunodeficiency virus (FIV) and avian sarcoma virus (ASV) [70,71,72,73,74,75]. It has also been suggested that MA is able to manipulate the lipid compositions of membranes. For instance, Gag accumulation at assembly sites on the PM has been shown to induce the formation of membrane microdomains known as lipid rafts [46,76,77]. This result was supported by other studies which indicated that viral membranes are typically enriched in sphingomyelin lipids and cholesterol, two major components of the lipid rafts [78]. Furthermore, it has been shown that MA is capable of generating PI(4,5)P_2_/cholesterol nanoclusters, possibly creating a positive feedback loop that drives lattice formation by enhancing the affinity of the Gag protein to the membrane [79,80]. In a recent study, analysis of HIV-1-infected cells revealed that, upon assembly, HIV-1 is able to specifically trap PI(4,5)P_2_ and cholesterol but not PE or sphingomyelin [81]. Data showed that Gag is the main driving force to restrict the mobility of PI(4,5)P_2_ and cholesterol at the PM. Other studies have shown that HIV-1 Gag binding to membranes is enhanced by inclusion of phosphatidylserine (PS) and cholesterol [15,27,28,44,58,82,83,84,85]. Additionally, RNA is considered a negative regulator of Gag−membrane interactions based on the studies that the MA domain of Gag was found to bind to specific tRNA in the cytosol [86], preventing Gag from interacting with intracellular membranes [23,24,58,87]. It was demonstrated that incorporation of PI(4,5)P_2_ into membranes inhibited the interaction with cellular RNA [23,24,58]. 

Over the past 15 years, we and others have used a variety of structural and biophysical approaches to characterize binding of retroviral MA proteins to phospholipids and membrane mimetics such as bicelles, micelles, liposomes and lipid nanodiscs (NDs) [42,70,71,72,73,74,88,89,90,91,92,93,94,95]. These studies provided invaluable insights on key molecular determinants of MA-mediated assembly. NMR studies revealed that binding of PI(4,5)P_2_ containing truncated (*tr*) acyl chains (*tr*-PI(4,5)P_2_) to HIV-1 MA induced a conformational change that promoted myr exposure [42]. The structure of MA bound to *tr*-PI(4,5)P_2_ showed that the 2’-acyl chain is inserted into a hydrophobic cleft, whereas the inositol group is packed against the HBR of MA (Figure 2) [42]. Interactions of HIV-1 Gag and MA with *tr*-PI(4,5)P_2_ have also been detected by mass spectrometry-based protein footprinting assays in which an NHS-biotin modification approach was used to identify the lysine residues protected from biotinylation by direct contact with *tr*-PI(4,5)P_2_ or tRNA [93]. The involvement of the acyl chain of PI(4,5)P_2_ in MA and Gag binding has been confirmed by surface plasmon resonance methods [92]. Subsequent structural studies on abundant PM lipids such as PS, phosphatidylcholine (PC) and phosphatidylethanolamine (PE) also containing truncated acyl chains, have shown that these three lipids bind to HIV-1 MA via a second distinct hydrophobic pocket on the protein [89]. Based on the NMR structural studies, models for HIV-1 MA binding to PI(4,5)P_2_-enriched membranes were proposed (Figure 2) [42,89]. Other models of membrane-bound HIV-1 MA were proposed based on NMR and biochemical studies with liposomes containing lipids with native acyl chains [91] or computational approaches [79]. These models suggested that acyl chains are not involved in MA binding, and that Gag–membrane interaction is mediated predominantly by dynamic, electrostatic interactions between the HBR and PI(4,5)P_2_/PS [91]. More recently, our laboratory utilized lipid NDs as a membrane mimetic to study binding of HIV-1 MA [95]. Lipid ND is a non-covalent assembly of phospholipids and a membrane scaffold protein (MSP) [96,97,98,99,100,101]. Phospholipids associate as a bilayer domain stabilized by two MSP molecules wrapped around the edges of the discoidal structure in a belt-like configuration. One advantage of using lipid NDs is that they can be modified in size and lipid composition. Another advantage is their ability to obtain quantitative measurements of binding to proteins. Studies from our laboratory revealed that up to ten molecules of MA are capable of binding to one ND, that the myr group is readily exposed and anchored to the membrane bilayer even in the absence of PI(4,5)P_2_, that the affinity of MA binding to NDs is enhanced upon incorporation of PS and PI(4,5)P_2_, and that the interaction interface in MA is located in the HBR [95]. These findings demonstrated that lipid NDs are relevant membrane mimetics to study binding of retroviral MA proteins. Altogether, a combination of structural, biochemical, biophysical, and computational approaches provided insights into the mechanisms by which the HIV-1 Gag polyproteins associate with the inner leaflet of the PM and facilitate virus assembly.

## 4. Structural Organization of MA on the Membrane

As mentioned above, a detailed macromolecular picture of MA arrangement on the membrane is still lacking. Cryo-electron reconstructions of immature virus particles and tubular CA assemblies have been used to obtain detailed structures of both the mature and immature CA and SP1 domains [29,102,103,104,105,106]. Whereas it is established that CA forms well-ordered hexamers in both mature and immature particles, studies were unable to provide details of the MA domain due to a lack of periodicity [29]. However, structural, biophysical and biochemical data indicate that MA can adopt a trimeric arrangement. The earliest indication that MA forms ordered oligomers is revealed in the trimeric x-ray structure of unmyristoylated MA (myr(–)MA) [38]. Sedimentation equilibrium and sedimentation velocity data indicated that, in solution, MA exists in a monomer–trimer equilibrium [41,51]. Cryo-electron diffraction data obtained from 2D crystals of MA grown on a lipid monolayer containing PI(4,5)P_2_ suggest that MA organizes as hexamers of trimers [107], providing further evidence that MA is capable of forming trimers on the membrane surface. 

**Figure 3:**
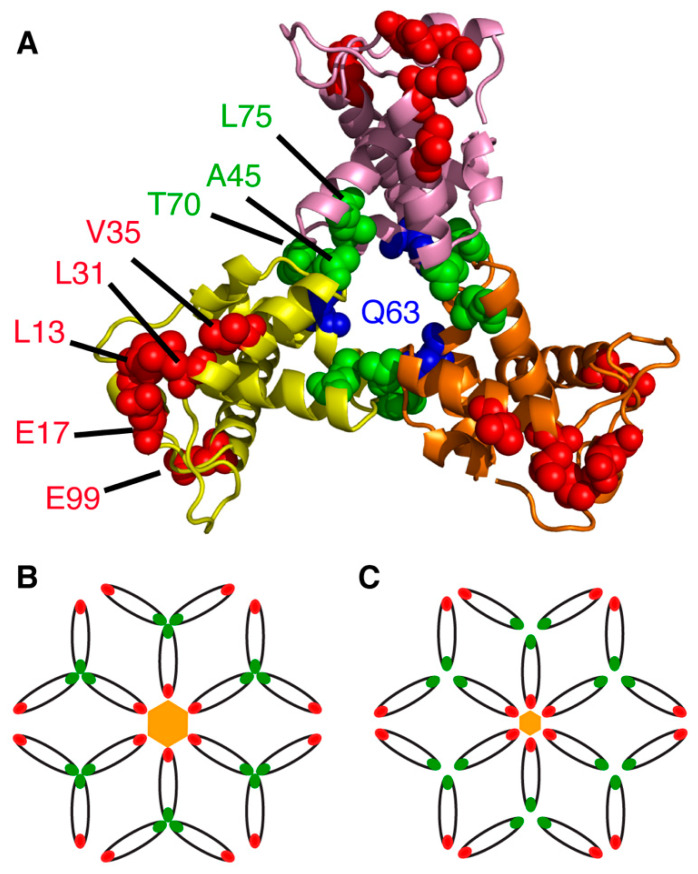
MA structural motifs that mediate Env incorporation. (**A**) Structure of the MA trimer (PDB ID: 1HIW) highlighting residues implicated in Env incorporation (red and green). The current hypothesis is that the revertant mutant Q63R enhances MA trimerization, thereby increasing MA affinity to gp41CT and enhancing Env incorporation. (**B**) Schematic representation of the hexamer of trimer arrangement of MA based on the models proposed Alfadhli et al. [107] and adopted by Freed et al. [108] to explain the mechanisms of Env incorporation. In this model, a ∼45 nm central aperture is formed by residues implicated in Env incorporation (red). It is hypothesized that the gp41CT protein is accommodated in the central aperture. Other mutations that were found to significantly disrupt Env incorporation are located in the trimer interface (green). (**C**) Based on an early model [110], perturbations of the putative hexameric or trimeric interface in the MA lattice are thought to create smaller central apertures (∼30 nm) that may cause a steric exclusion of gp41CT.

Analysis of the x-ray structure of the myr(–)MA protein (PDB ID: 1HIW) show that the side chain of Gln^63^ in one molecule is less than 5 Å from the side chain of Ser^66^ in an adjacent molecule (Figure 3), a distance deemed adequate for chemical cross-linking. Freed and co-workers provided genetic evidence for an interaction between these two residues within the Gag polyprotein in cells infected with HIV-1. Substitution of Gln^63^ and Ser^66^ to lysines had no significant impact on virus replication [108]. In a cross-linking assay using glutaraldehyde in Jurkat and MT4 cells, MA dimers and trimers were observed [108]. These findings were further complemented by amino acid substitutions to assess the impact of manipulating the trimer interface on virus replication (discussed below). Until recently, structural details of the MA trimer in solution were not defined because of the lack of a stable recombinant construct that capitulates the functional trimer protein. To structurally characterize the MA trimer in solution, our laboratory engineered a stable recombinant MA trimer construct by fusing the foldon domain (FD) of phage T4 fibritin to the MA C terminus [95]. NMR data confirmed that attachment of FD did not alter the structure of the MA protein. Employing hydrogen-deuterium exchange MS (HDX-MS), we identified an MA–MA interface in the MA-foldon trimer that is similar to that observed in the x-ray structure of the myr(–)MA protein [38]. We have also established that trimerization dramatically enhances the affinity of the MA domain to lipid bilayers [95]. Altogether, these results are in line with the observation that PM association of MA is enhanced in the context of the immature, uncleaved Gag protein [44,45,49,109], and further validates previous findings that MA is capable of forming trimers on membranes [107].

## 5. Structure and Function of the Envelope Glycoprotein

**Figure 4:**
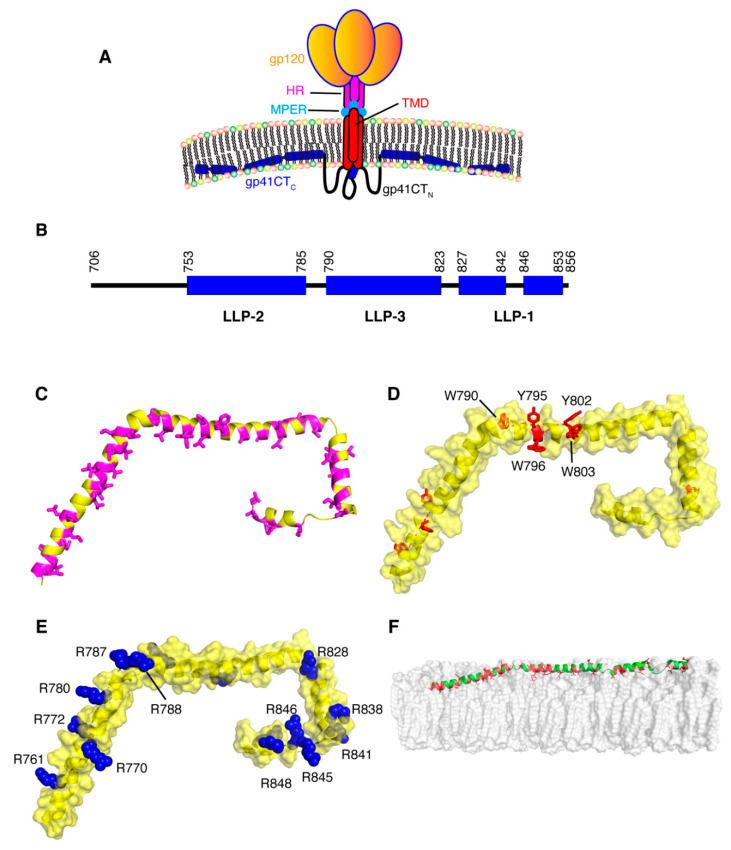
Structural features of the gp41CT protein. (**A**) A model depicting the gp120 and gp41 proteins on the surface of an HIV-1 particle. (**B**) Schematic representation of the gp41CT protein sequence with secondary structure illustration. (**C**) Cartoon representation of gp41CT_C_ showing the extensive hydrophobic interface formed by Leu, Ile, Val, Ala, Trp and Phe residues (magenta). (**D**) Surface representation of gp41CT_C_ showing aromatic residues as red sticks. Of particular interest is the cluster of aromatic residues at the beginning of LLP3, which harbors the Y795/W796 motif implicated in Env incorporation [114,115,116]. The majority of aromatic residues are buried in the interior of membrane. (**E**) Surface representation of the gp41CT_C_ protein showing Arg and Lys residues (blue). (**F**) A model of gp41CT_C_ bound to a membrane bilayer. Residues colored in red interact extensively with the interior of the membrane. Membrane bilayer was generated in VMD membrane builder plug-in [53].

For decades, research on the Env protein has been predominantly focused on elucidating the mechanisms of host–receptor binding, membrane fusion and evasion of recognition by the immune system. The viral surface protein Env is synthesized in the rough endoplasmic reticulum (RER) and subsequently trafficked to the PM through the secretory pathway for incorporation into budding virus particles (Figure 1) [111]. Env is a highly glycosylated, membrane-spanning protein which facilitates cell infection through surface receptor binding and membrane fusion. Env is initially synthesized as gp160, which forms trimers prior to exiting the ER [112]. This is followed by passage through the Golgi network, during which gp160 undergoes heavy glycosylation and is cleaved by furin or furin-like proteases to produce the surface glycoprotein gp120 and the transmembrane protein gp41 [111,113]. The two subunits remain non-covalently bound in a trimer of heterodimers arrangement that protrudes from the virion surface as glycosylated Env spikes [111,113]. The gp120 subunits are localized entirely on the exterior of the virus particle and contain the highly conserved cell receptor and co-receptor binding sites (CD4 and CXCR4/CCR5 respectively) [111,113]. By virtue of its structure, the magnitude of glycosylation and high levels of sequence variability, gp120 is uniquely adept at evasion of the host immune response [111,113]. The gp41 subunit consists of a fusogenic ectodomain, a transmembrane domain (TMD) and the CT (Figure 4).

Receptor and co-receptor binding by gp120 initiate large conformational changes in both subunits, bringing a fusion domain into play. The fusion motif in gp41 is composed of a hydrophobic peptide and two heptad repeats (HR) that fold into a six-helix bundle during fusion [113,117,118]. This causes a destabilization of the membrane followed by a series of conformational changes in gp41, bringing the two membranes to a fusion state [113]. A short peptide preceding the TMD is called the membrane proximal external region (MPER) and is considered a key target for broadly neutralizing monoclonal antibodies (Figure 4) [113,119,120,121]. The MPER, which residues outside the lipid bilayer, folds into a threefold cluster stabilized by conserved hydrophobic residues and potentially by an interaction with phospholipid headgroups [122]. The TMD consists of a single hydrophobic α-helix from each gp41 subunit, which associate into a trimeric helical bundle [123,124,125]. In other studies it has been indicated that the TMD may exist in a monomeric confirmation, suggesting that trimerization could be driven by the ectodomain [126,127,128]. Located on the cytoplasmic side of the membrane, downstream from the TMD, is gp41CT (Figure 1). For lentiviruses, gp41CT is unusually long (~150 amino acids) compared to other members of the *Retroviridae* family (~20–30 amino acids) [129]. In general, expendable genetic information is quickly discarded in lentiviruses [130,131]. Consequently, it is not fully understood why certain lentiviruses appropriate valuable genetic space to such long CTs when similar viruses are able to function properly in their absence [111]. It has been shown that HIV-1 gp41CT contains motifs that interact with cellular components, implicating its involvement in a variety of functions [129]. These interacting partners include CaM, which was shown to play a role in apoptosis [132,133]; clathrin adaptor proteins AP-1 and AP-2, which are responsible for endocytosis of Env and are thereby involved in controlling Env cell surface concentrations [134,135,136,137]; and Rab11-family interacting protein 1C (FIP1C), an endosomal trafficking complex required for Env incorporation in nonpermissive cell lines [114,115,116]. Separate from these functions, there is substantial evidence that gp41CT, through an interaction with the MA domain of Gag, plays a critical role in Env incorporation into virus particles (discussed below) [138,139,140].

## 6. Structure and Topology of gp41CT

Structural and functional models of gp41CT have often relied on primary sequence analysis and biophysical characterization of short peptide fragments derived from the gp41CT protein [141,142,143,144,145]. The gp41CT domain has long been characterized by the presence of three amphipathic α-helical segments, referred to as lentivirus lytic peptide 1 (LLP-1), LLP-2 and LLP-3, which are highly conserved not only among HIV-1 strains but also among HIV-2, simian immunodeficiency virus and equine infectious anemia virus [146,147,148]. LLP-1 and LLP-2 were shown to be inserted into viral membranes by a photoinduced chemical reactions [149]. These LLP motifs have also been implicated in a variety of functions, such as cell surface expression [150], Env fusogenicity [151] and Env protein stability [152], as well as Env incorporation into budding particles [140,153]. Until recently, production of significant quantities of stable recombinant gp41CT proteins and reconstitution in a membrane mimetic have been a barrier to obtaining detailed structural information on the protein. Our laboratory determined the structure of gp41CT by NMR methods and characterized its interaction with membranes [154]. It has been shown that the N-terminal region of gp41CT (gp41CT_N_, residues 707–751) lacks an ordered secondary structure and has no propensity for membrane interaction. However, the C-terminal domain (gp41CT_C_, residues 752–856) consists of three consecutive amphipathic α-helices (LLP2, LLP3 and LLP1) and is tightly associated with the membrane (Figure 4) [154]. Structural data also revealed a variable degree of membrane penetration among the three helices with the N-terminal LLP2 helix penetrating deeper than LLP3 and LLP1 (Figure 4). The helical structures of LLP2 and LLP3 contain several cation-π interactions between aromatic and basic residues in the *i* and *i*+4 positions, respectively. The structural findings do not support models that postulate the existence of a membrane-spanning domain within gp41CT_N_, which are based on primary sequence analysis and the existence of the highly immunogenic epitope region (Kennedy epitope, KE) within gp41CT [142,155,156]. However, it is possible that the KE may become exposed to the exterior of the viral membrane during periods of membrane disruption such as the fusion process, but that exposure is unlikely under stable conditions. As previously noted, [148] gp41CT_C_ contains a number of highly conserved arginines within the LLP1 and LLP2 motifs (Figure 4), many of which are involved in cation-π interactions. Selective incorporation and conservation of arginines over lysines in these motifs is not fully understood. It was reported that mutations of arginine to lysine in the LLP1 motif resulted in significant impairment of Env expression and consequently virus replication kinetics, Env fusogenicity and incorporation. In contrast, Arg-to-Lys substitutions in LLP2 only affected the level of Env incorporation and fusogenicity [157]. Altogether, the structural details on gp41CT [154] have provided insights that may help to understand the mechanisms of Gag-mediated Env incorporation as well as the overall Env mobility and conformation on the virion surface.

## 7. Mechanisms of Env Incorporation

Early studies have shown that gp41CT plays a functional role in Env incorporation and virus replication in T-cell lines (CEM, Jurkat, and MT-2), phytohemagglutinin (PHA)-stimulated peripheral blood mononuclear cells (PBMCs) and monocyte-derived macrophages (MDMs) [158,159]. However, permissive cells such as 293T cells and semi-permissive cells (HeLa and MT-4) allow passive incorporation of Env in a CT-independent manner. These results strongly supported a role for host cell factors that may differ between cell types. Studies by Spearman and co-workers demonstrated that FIP1C is required for CT-dependent incorporation of Env into HIV-1 particles [114,115,116]. FIPs are effectors of Rab11 GTPases that mediate a sorting of cargo from the endosomal recycling compartment to the PM [160]. The requirement of Rab11-FIP1C for the trafficking of Env from the endosomal recycling compartment to the PM likely explains the cell-type dependent nature of the Env incorporation defects imposed by gp41CT deletions [114,115,116]. Of note, the Y795/W796 motif in gp41CT was identified as a critical determinant for CT-dependent FIP1C redistribution out of the endosomal recycling compartment and subsequent Env incorporation into virus particles [114,115,116]. Substitution of Y795/W796 into Ser-Leu completely recreated the pattern of cell-type dependence on Env incorporation previously observed with CT truncation, and FIP1C depletion had no effect on the level of incorporation of this mutant Env. These results suggested that Y795/W796 and FIP1C mediate Env incorporation in a cell-type-specific manner. Interestingly, structural studies have revealed that W796 is located on the hydrophobic, membrane-interacting side of the LLP3 helix whereas Y795 is localized on the exposed polar side (Figure 4) [154]. It has yet to be established at the molecular level whether an interaction occurs between the Rab11-FIP1C complex and gp41CT during Env trafficking. 

After trafficking through the trans-Golgi network in an apparent FIP1C-dependent manner, the mature Env proteins are deposited onto the PM where they eventually make their way to sites of Gag assembly for incorporation into virus particles [111]. This phenomenon is documented by several studies. Early high-resolution confocal imaging studies have shown that Gag and Env colocalize at the PM and that mistargeting of a mutant Gag to the Golgi apparatus alters the pattern of surface expression of Env [55]. Other studies have shown that Gag assembly induced the aggregation of small Env clusters into larger domains that were completely immobile [161]. Truncation of gp41CT abrogated Gag’s ability to induce Env clustering and restored Env mobility at assembly sites. Super-resolution microscopy data also indicated that recruitment of HIV-1 Env to viral assembly sites is dependent on gp41CT [162]. By interrogating the subviral angular distribution of Env on the cell-associated virus using multicolor three-dimensional super-resolution microscopy, it was recently demonstrated that, in a manner dependent on cell-type and on the full-length gp41CT, the distribution of Env is biased toward the necks of cell-associated particles [163]. It was postulated that the neck-biased distribution is regulated by vesicular retention and steric complementarity of Env during independent Gag lattice formation [163]. Altogether, these studies indicated a functional role of gp41CT in Gag–Env co-localization and Env incorporation.

Although the mechanism of Env incorporation is still not fully understood, genetic and biochemical data indicates that an interplay between the MA domain of Gag and gp41CT exists. In earlier studies, HIV-1 has been shown to exhibit a specific basolateral release in polarized epithelial cells [164,165]. In other studies investigating polarized HIV-1 viral budding following introduction of proviral DNA constructs, it was shown that the targeting signal for polarized virus release in part localized within the gp41CT [166]. Mutants of the MA domain of Gag were shown to be nonpolarized only when unable to interact with Env. These results are consistent with a model of polarized virus budding in which the Gag proteins are targeted for specific basolateral release via an interaction of MA with the CT of Env. Other studies have identified a triple MA mutant (L21K/E74K/A83T) that blocks the infectivity of HIV-1 particles pseudotyped with MLV Env glycoproteins without affecting infectivity conferred by HIV-1 Env or vesicular stomatitis virus G glycoproteins [167]. However, this triple mutant does not affect the incorporation of MLV Env into virions. It was also demonstrated that in HIV-1 virions pseudotyped with MLV Env, the HIV-1 protease (PR) efficiently catalyzes the cleavage of the p15(E) transmembrane (TM) protein to a 12-kDa protein (p12(E)) and a 16-residue peptide (p2(E) or R); the MA mutant, however, blocked this HIV-1 PR-mediated cleavage of MLV TM. Additionally, it was shown the transdominant inhibition exerted by the mutant MA on infectivity correlated with the relative level of p15(E) cleavage, and that mutant virions are significantly more infectious when pseudotyped with a truncated p12(E) form of MLV Env. Taken together, these results indicate that HIV-1 Gag can influence the viral PR-mediated processing of the MLV TM Env protein p15(E). More recently, Env incorporation was analyzed in pseudotyped HIV-1 and MLV particles [168]. It was found that one form of MLV Env is compatible with MLV particles but incompatible with HIV-1 particles, while a second form is compatible with HIV-1 particles but not with MLV particles. The HIV-1 particle incompatibility correlated with a less efficient cleavage of the R peptide; however, the MLV particle incompatibility was subtle. It was suggested that MLV incompatibility is caused by a lack of incorporation into particles, yet incorporation could be restored by further truncation of the CT or by using a chimeric MLV Gag protein containing the HIV-1 MA without fully restoring infectivity [168].

Other studies have shown that deletions and point mutations in MA (L13E, E17K, L31E, V35E and E99V) have adverse effects on Env incorporation without affecting virus particle formation, suggesting that an interaction with MA was perhaps required [169,170,171,172,173]. Analysis of MA trimer formation was expanded through a series of conservative and nonconservative mutations at, or near, the trimer interface in the crystal structure (Figure 3). Whereas several polar residues that are sufficiently close together to potentially form hydrogen bonds at the trimer interface (Asn^47^, Gln^58^ and Gln^68^) did not seem to contribute to trimer formation, nonconservative mutations (A45E, T70R, L75G and L75E) inhibited MA trimerization, as demonstrated using a cross-linking assay, yielding particles impaired for Env incorporation and infectivity [108]. These results suggested that a small cluster of hydrophobic interactions appear to be required for the formation of the MA trimer [108]. Additionally, point mutations within gp41CT, as well as deletion of the tail entirely, resulted in a similar phenotype of Env-deficient virus particles in non-permissive cell types, indicating that an interaction between MA and gp41CT likely mediated this process [170,172]. Env incorporation defects caused by mutations in MA could be completely reversed by a compensatory amino acid substitution in MA (Q63R) [174], indicating an interplay between the membrane-bound MA and gp41CT in regulating Env incorporation. 

**Figure 5:**
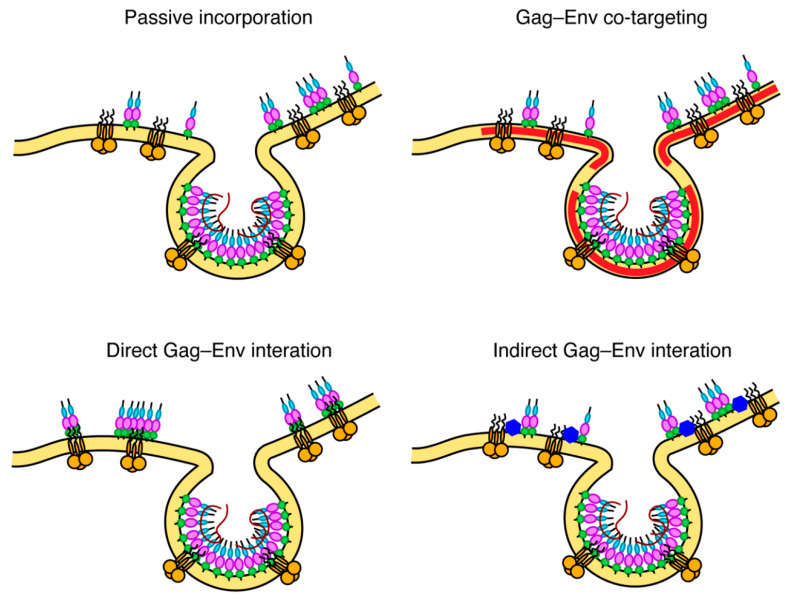
Possible models for Env incorporation. The passive model assumes that Env is randomly incorporated as particles may acquire a host-derived membrane during assembly and budding. The Gag–Env co-targeting model suggests that both proteins are recruited to assembly sites based on their independent association with an element of the PM such as lipid raft-like microdomains. The direct Gag–Env interaction model postulates that an interaction occurs between the MA domain of the Gag lattice and gp41CT. The indirect interaction model suggests that interaction is mediated by a cellular factor. These models were first proposed by Tedbury et al. [111].

To explain the molecular basis for Env incorporation, a number of models have been proposed (Figure 5) [111]. These models, which are not mutually exclusive, are divided into passive incorporation, Gag–Env co-targeting, direct Gag–Env interaction and indirect Gag–Env interaction. The passive model assumes that Env is randomly incorporated as the particles acquire host-derived membranes during assembly and budding. The Gag–Env co-targeting model suggests that both proteins are recruited to assembly sites based on their independent association with an element of the PM such as lipid raft-like microdomains. The Gag–Env interaction model postulates that an interaction occurs between the two proteins (either directly or mediated by a cellular factor) and that this interaction facilitates both the recruitment of Env to the assembly sites and its incorporation into the Gag lattice [111]. An interaction between gp41CT and MA is also supported by the fact that Env is non-fusogenic in immature virus particles and only becomes active upon Gag cleavage. Fusion activity can also be triggered by truncation of gp41CT, suggesting that Env is locked in a non-fusogenic conformation by an interaction with uncleaved Gag [175,176]. Nanoscale single particle tracking of Env on the PM has demonstrated that Env immobilization at sites of Gag assembly requires gp41CT and Leu^13^ of MA but does not require the curvature of the lattice [177]. This same study also showed that Env was restricted to subviral regions within the Gag lattice, indicating that an interaction between gp41CT and MA may be responsible for Env retention in budding particles. While it is strongly suggested that an interaction between gp41CT and MA is involved, the exact nature of this interaction (whether by direct or indirect interactions, or by steric trapping) is still unclear. A few reports have suggested direct interaction between MA and gp41CT based on glutathione S-transferase (GST) pull-down assays [173,178]. Regardless, the fact that the MA mutations allowed for the incorporation of the short but not the long tailed Env in certain cell types may suggest that MA mutants may confer a steric hindrance preventing the accommodation of the full-length gp41CT. 

Freed and co-workers have provided biochemical evidence that MA trimerization is an obligatory step in the assembly of infectious HIV-1 and demonstrated a correlation between loss of MA trimerization and loss of Env incorporation [108]. By analysis of the hexamer of trimer model of MA, it was noted that residues which appeared to be essential for Env incorporation (Leu^13^, Glu^17^, Leu^31^, Val^35^ and Glu^99^) point toward the hexamer centers (Figure 3), which led to the hypothesis that gp41CT may interact with these residues [107,110,179]. It is noted that residue Gln^63^, which suppressed Env incorporation defects elicited by the L13E, E17K, L31E, V35E and E99V MA mutations and of an gp41CT mutation with the same phenotype [108,138,139,180], is located at the trimer interface (Figure 3). The current prevailing model suggests that Q63R mutation may stabilize the trimer structure such that MA lattices, which form large hexamer holes, are favored over those that feature smaller hexamer holes. This model is supported by biochemical evidence that Q63R mutation modestly enhanced MA trimer and promoted interaction with gp41CT without altering the organization of MA on a membrane layer [178,181]. By serially propagating MA trimerization-defective mutants in T cell lines, compensatory mutations that rescue MA trimer interface mutants with severely impaired Env incorporation were identified [179]. The compensatory mutations that are located within or near the trimer interface and in other distant regions, restored Env incorporation as well as particle infectivity and permitted replication in culture. Altogether, the current hypothesis is that revertant mutants such as Q63R likely contribute to MA trimerization, increase affinity to gp41CT and enhance Env incorporation. However, this hypothesis has yet to be tested at the structural level.

## 8. Gag–Env–Membrane Complex as a Therapeutic Target

Our understanding of the primary mechanism of Env incorporation in HIV-1 has been greatly expanded over the past decade. However, there still exist major gaps in our knowledge. We believe that high-resolution structural data on a biologically relevant complex between gp41CT and MA in the context of a lipid bilayer can be crucial to this endeavor. As a key player in HIV-1 assembly and maturation, HIV-1 Gag and its individual domains emerged as attractive drug targets [182,183]. Recent advances in the development of potent CA and maturation (CA-SP1 cleavage site) inhibitors have demonstrated the therapeutic viability of Gag domains ([182,183] and references therein). Herein, we discussed three key features in viral assembly that could potentially serve as targets for anti-retroviral drugs: MA–membrane interface, MA trimer and gp41CT–MA interface. Small molecule inhibitors that target the PI(4,5)P_2_ binding site in MA have been identified [184,185,186]. Using an SPR assay with immobilized biotinylated PI(4,5)P_2_, it was shown that these inhibitors are able to compete with PI(4,5)P_2_ [185,186]. Our lab has developed NMR– and ITC–based assays to investigate MA binding to lipid NDs enriched with PI(4,5)P_2_ [95]. These assays could be utilized to screen or design small molecules that are capable of disrupting the MA–membrane interactions. The discovery that Env incorporation is dependent on MA trimerization renders the MA–MA interface an attractive potential target for small molecule or peptide-based inhibitors, a task that has proved to be elusive due to the lack of a stable recombinant MA trimer. We hope that our stable and native-like MA-foldon construct and the application of HDX-MS techniques can serve as innovative tools to develop inhibitors that target the MA–MA interface. Lastly, the enigmatic gp41CT–MA interaction could potentially serve as a novel target for inhibitory drugs. In summary, an abundance of genetic and biochemical data, combined with the structural data on gp41CT and MA trimer, may provide insights into the best path toward detailed structural characterization of the gp41CT–MA–membrane complex, a key step in the pursuit of novel antiviral drugs that target virus assembly and Env incorporation.

## References

[1] Rodger A.J., Lodwick R., Schechter M., Deeks S., Amin J., Gilson R., Paredes R., Bakowska E., Engsig F.N., Phillips A. (2013). Mortality in well controlled HIV in the continuous antiretroviral therapy arms of the SMART and ESPRIT trials compared with the general population. AIDS.

[2] Cohen M.S., Chen Y.Q., McCauley M., Gamble T., Hosseinipour M.C., Kumarasamy N., Hakim J.G., Kumwenda J., Grinsztejn B., Pilotto J.H. (2016). Antiretroviral therapy for the prevention of HIV-1 transmission. N. Engl. J. Med..

[3] Arts E.J., Hazuda D.J. (2012). HIV-1 antiretroviral drug therapy. Cold Spring Harb. Perspect. Med..

[4] Duarte H.A., Panpradist N., Beck I.A., Lutz B., Lai J., Kanthula R.M., Kantor R., Tripathi A., Saravanan S., MacLeod I.J. (2017). Current Status of Point-of-Care Testing for Human Immunodeficiency Virus Drug Resistance. J. Infect. Dis..

[5] Menendez-Arias L. (2013). Molecular basis of human immunodeficiency virus type 1 drug resistance: Overview and recent developments. Antivir. Res..

[6] Shukla E., Chauhan R. (2019). Host-HIV-1 Interactome: A Quest for Novel Therapeutic Intervention. Cells.

[7] Engelman A., Cherepanov P. (2012). The structural biology of HIV-1: Mechanistic and therapeutic insights. Nat. Rev. Microbiol..

[8] Houck-Loomis B., Durney M.A., Salguero C., Shankar N., Nagle J.M., Goff S.P., D’Souza V.M. (2011). An equilibrium-dependent retroviral mRNA switch regulates translational recoding. Nature.

[9] Jacks T., Power M.D., Masiarz F.R., Luciw P.A., Barr P.J., Varmus H.E. (1988). Characterization of ribosomal frameshifting in HIV-1 gag-pol expression. Nature.

[10] Mucksch F., Laketa V., Muller B., Schultz C., Krausslich H.G. (2017). Synchronized HIV assembly by tunable PIP_2_ changes reveals PIP_2_ requirement for stable Gag anchoring. eLife.

[11] Hendrix J., Baumgartel V., Schrimpf W., Ivanchenko S., Digman M.A., Gratton E., Krausslich H.G., Muller B., Lamb D.C. (2015). Live-cell observation of cytosolic HIV-1 assembly onset reveals RNA-interacting Gag oligomers. J. Cell Biol..

[12] Gousset K., Ablan S.D., Coren L.V., Ono A., Soheilian F., Nagashima K., Ott D.E., Freed E.O. (2008). Real-time visualization of HIV-1 GAG trafficking in infected macrophages. PLoS Pathog..

[13] Jouvenet N., Neil S.J.D., Bess C., Johnson M.C., Virgen C.A., Simon S.M., Bieniasz P.D. (2006). Plasma membrane is the site of productive HIV-1 particle assembly. PLoS Biol..

[14] Welsch S., Keppler O.T., Habermann A., Allespach I., Krijnse-Locker J., Kräusslich H.-G. (2007). HIV-1 buds predominantly at the plasma membrane of primary human macrophages. PLoS Pathog..

[15] Chukkapalli V., Hogue I.B., Boyko V., Hu W.-S., Ono A. (2008). Interaction between HIV-1 Gag matrix domain and phosphatidylinositol-(4,5)-bisphosphate is essential for efficient Gag-membrane binding. J. Virol..

[16] Ono A., Ablan S.D., Lockett S.J., Nagashima K., Freed E.O. (2004). Phosphatidylinositol (4,5) bisphosphate regulates HIV-1 Gag targeting to the plasma membrane. Proc. Natl. Acad. Sci. USA.

[17] Olety B., Ono A. (2014). Roles played by acidic lipids in HIV-1 Gag membrane binding. Virus Res..

[18] Freed E.O. (2015). HIV-1 assembly, release and maturation. Nat. Rev. Microbiol..

[19] D’Souza V., Summers M.F. (2005). How retroviruses select their genomes. Nat. Rev. Microbiol..

[20] Rein A. (2019). RNA Packaging in HIV. Trends Microbiol..

[21] Ganser-Pornillos B.K., Yeager M., Sundquist W.I. (2008). The structural biology of HIV assembly. Curr. Opin. Struct. Biol..

[22] Sundquist W.I., Kräusslich H.-G. (2012). HIV-1 assembly, budding, and maturation. Cold Spring Harb. Perspect. Med..

[23] Chukkapalli V., Inlora J., Todd G.C., Ono A. (2013). Evidence in support of RNA-mediated inhibition of phosphatidylserine-dependent HIV-1 Gag membrane binding in cells. J. Virol..

[24] Chukkapalli V., Ono A. (2011). Molecular Determinants that Regulate Plasma Membrane Association of HIV-1 Gag. J. Mol. Biol..

[25] Purohit P., Dupont S., Stevenson M., Green M.R. (2001). Sequence-specific interaction between HIV-1 matrix protein and viral genomic RNA revealed by in vitro genetic selection. RNA.

[26] Li H., Dou J., Ding L., Spearman P. (2007). Myristoylation is required for human immunodeficiency virus type 1 Gag-Gag multimerization in mammalian cells. J. Virol..

[27] Dalton A.K., Ako-Adjei D., Murray P.S., Murray D., Vogt M.V. (2007). Electrostatic Interactions Drive Membrane Association of the Human Immunodeficiency Virus Type 1 Gag MA Domain. J. Virol..

[28] Dick R.A., Goh S.L., Feigenson G.W., Vogt V.M. (2012). HIV-1 Gag protein can sense the cholesterol and acyl chain environment in model membranes. Proc. Natl. Acad. Sci. USA.

[29] Wright E.R., Schooler J.B., Ding H.J., Kieffer C., Fillmore C., Sundquist W.I., Jensen G.J. (2007). Electron crytomography of immature HIV-1 virions reveals the structure of the CA and SP1 Gag shells. EMBO J..

[30] Briggs J.A., Riches J.D., Glass B., Bartonova V., Zanetti G., Krausslich H.G. (2009). Structure and assembly of immature HIV. Proc. Natl. Acad. Sci. USA.

[31] Ganser-Pornillos B.K., Yeager M., Pornillos O. (2012). Assembly and architecture of HIV. Adv. Exp. Med. Biol..

[32] Votteler J., Sundquist W.I. (2013). Virus budding and the ESCRT pathway. Cell Host Microbe.

[33] Lippincott-Schwartz J., Freed E.O., van Engelenburg S.B. (2017). A Consensus View of ESCRT-Mediated Human Immunodeficiency Virus Type 1 Abscission. Annu. Rev. Virol..

[34] Pornillos O., Ganser-Pornillos B.K. (2019). Maturation of retroviruses. Curr. Opin. Virol..

[35] Massiah M.A., Starich M.R., Paschall C., Summers M.F., Christensen A.M., Sundquist W.I. (1994). Three dimensional structure of the human immunodeficiency virus type 1 matrix protein. J. Mol. Biol..

[36] Matthews S., Barlow P., Boyd J., Barton G., Russell R., Mills H., Cunningham M., Meyers N., Burns N., Clark N. (1994). Structural similarity between the p17 matrix protein of HIV-1 and interferon-g. Nature (London).

[37] Matthews S., Barlow P., Clark N., Kingsman S., Kingsman A., Campbell I. (1995). Refined solution structure of p17, the HIV matrix protein. Biochem. Soc. Trans..

[38] Hill C.P., Worthylake D., Bancroft D.P., Christensen A.M., Sundquist W.I. (1996). Crystal Structures of the Trimeric HIV-1 Matrix Protein: Implications for Membrane Association. Proc. Natl. Acad. Sci. USA.

[39] Massiah M.A., Worthylake D., Christensen A.M., Sundquist W.I., Hill C.P., Summers M.F. (1996). Comparison of the NMR and X-ray structures of the HIV-1 matrix protein: Evidence for conformational changes during viral assembly. Protein Sci..

[40] Tang C., Ndassa Y., Summers M.F. (2002). Structure of the N-terminal 283-residue fragment of the immature HIV-1 Gag polyprotein. Nat. Struct. Biol..

[41] Tang C., Loeliger E., Luncsford P., Kinde I., Beckett D., Summers M.F. (2004). Entropic switch regulates myristate exposure in the HIV-1 matrix protein. Proc. Natl. Acad. Sci. USA.

[42] Saad J.S., Miller J., Tai J., Kim A., Ghanam R.H., Summers M.F. (2006). Structural basis for targeting HIV-1 Gag proteins to the plasma membrane for virus assembly. Proc. Natl. Acad. Sci. USA.

[43] Bryant M., Ratner L. (1990). Myristoylation-Dependent Replication and Assembly of Human Immunodeficiency Virus 1. Proc. Natl. Acad. Sci. USA.

[44] Zhou W., Resh M.D. (1996). Differential membrane binding of the human immunodeficiency virus type 1 matrix protein. J. Virol..

[45] Spearman P., Horton R., Ratner L., Kuli-Zade I. (1997). Membrane binding of human immunodeficiency virus type 1 matrix protein in vivo supports a conformational myristyl switch mechanism. J. Virol..

[46] Nguyen D.H., Hildreth J.E. (2000). Evidence for budding of human immunodeficiency virus type 1 selectively from glycolipid-enriched membrane lipid rafts. J. Virol..

[47] Provitera P., El-Maghrabi R., Scarlata S. (2006). The effect of HIV-1 Gag myristoylation on membrane binding. J. Mol. Biol..

[48] Hermida-Matsumoto L., Resh M.D. (1999). Human immunodeficiency virus type 1 protease triggers a myristoyl switch that modulates membrane binding fo Pr55gag and p17MA. J. Virol..

[49] Paillart J.-C., Gottlinger H.G. (1999). Opposing effects of human immunodeficiency virus type 1 matrix mutations support a myristyl switch model of Gag membrane targeting. J. Virol..

[50] Ono A., Freed E.O. (1999). Binding of Human Immunodeficiency Virus Type 1 gag to membrane: Role of the matrix amino terminus. J. Virol..

[51] Fledderman E.L., Fujii K., Ghanam R.H., Waki K., Prevelige P.E., Freed E.O., Saad J.S. (2010). Myristate exposure in the human immunodeficiency virus type 1 matrix protein is modulated by pH. Biochemistry.

[52] Ghanam R.H., Fernandez T.F., Fledderman E.L., Saad J.S. (2010). Binding of calmodulin to the HIV-1 matrix protein triggers myristate exposure. J. Biol. Chem..

[53] Humphrey W., Dalke A., Schulten K. (1996). VMD: Visual molecular dynamics. J. Mol. Graph..

[54] Hanwell M.D., Curtis D.E., Lonie D.C., Vandermeersch T., Zurek E., Hutchison G.R. (2012). Avogadro: An advanced semantic chemical editor, visualization, and analysis platform. J. Cheminform..

[55] Hermida-Matsumoto L., Resh M.D. (2000). Localization of Human Immunodeficiency virus Type 1 Gag and env at the Plasma Membrane by Confocal Imagine. J. Virol..

[56] Ono A., Demirov D., Freed E.O. (2000). Relationship between human immunodeficiency virus Type-1 Gag multimerization and membrane binding. J. Virol..

[57] Ono A., Freed E.O. (2004). Cell-Type-Dependent Tageting of Human Immunodeficiency Virus Type 1 Assembly to the Plasma Membrane and the Multivesicular body. J. Virol..

[58] Chukkapalli V., Oh S.J., Ono A. (2010). Opposing mechanisms involving RNA and lipids regulate HIV-1 Gag membrane binding through the highly basic region of the matrix domain. Proc. Natl. Acad. Sci. USA.

[59] Hammond G.R., Fischer M.J., Anderson K.E., Holdich J., Koteci A., Balla T., Irvine R.F. (2012). PI4P and PI(4,5)P2 are essential but independent lipid determinants of membrane identity. Science.

[60] Kolay S., Basu U., Raghu P. (2016). Control of diverse subcellular processes by a single multi-functional lipid phosphatidylinositol 4,5-bisphosphate [PI(4,5)P2]. Biochem. J..

[61] Behnia R., Munro S. (2005). Organelle identity and the signposts for membrane traffic. Nature.

[62] McLaughlin S., Murray D. (2005). Plasma membrane phosphoinositide organization by protein electrostatics. Nature.

[63] McLaughlin S., Wang J., Gambhir A., Murray D. (2002). PIP2 and Proteins: Interactions, Organization, and Information Flow. Annu. Rev. Biophys. Biomol. Struct..

[64] Simonsen A., Wurmser A.E., Emr S.D., Stenmark H. (2001). The role of phosphoinositides in membrane transport. Curr. Opin. Cell Biol..

[65] Wenk M.R., De Camilli P. (2004). Protein-lipid interactions and phosphoinositide metabolism in membrane traffic: Insights from vesicle recycling in nerve terminals. Proc. Natl. Acad. Sci. USA.

[66] Agamasu C., Ghanam R.H., Xu F., Sun Y., Chen Y., Saad J.S. (2017). The Interplay between Calmodulin and Membrane Interactions with the Pleckstrin Homology Domain of Akt. J. Biol. Chem..

[67] Feng J., He L., Li Y., Xiao F., Hu G. (2019). Modeling of PH Domains and Phosphoinositides Interactions and Beyond. Adv. Exp. Med. Biol..

[68] Hurley J.H., Meyer T. (2001). Subcellular targeting by membrane lipids. Curr. Opin. Cell Biol..

[69] Yamamoto E., Kalli A.C., Yasuoka K., Sansom M.S. (2016). Interactions of Pleckstrin Homology Domains with Membranes: Adding Back the Bilayer via High-Throughput Molecular Dynamics. Structure.

[70] Stansell E., Apkarian R., Haubova S., Diehl W.E., Tytler E.M., Hunter E. (2007). Basic residues in the Mason-Pfizer monkey virus gag matrix domain regulate intracellular trafficking and capsid-membrane interactions. J. Virol..

[71] Hamard-Peron E., Juillard F., Saad J.S., Roy C., Roingeard P., Summers M.F., Darlix J.L., Picart C., Muriaux D. (2010). Targeting of murine leukemia virus gag to the plasma membrane is mediated by PI(4,5)P_2_/PS and a polybasic region in the matrix. J. Virol..

[72] Prchal J., Srb P., Hunter E., Ruml T., Hrabal R. (2012). The Structure of Myristoylated Mason-Pfizer Monkey Virus Matrix Protein and the Role of Phosphatidylinositol-(4,5)-Bisphosphate in Its Membrane Binding. J. Mol. Biol..

[73] Saad J.S., Ablan S.D., Ghanam R.H., Kim A., Andrews K., Nagashima K., Soheilian F., Freed E.O., Summers M.F. (2008). Structure of the myristylated HIV-2 MA protein and the role of phosphatidylinositol-(4,5)-bisphosphate in membrane targeting. J. Mol. Biol..

[74] Brown L.A., Cox C., Baptiste J., Summers H., Button R., Bahlow K., Spurrier V., Kyser J., Luttge B.G., Kuo L. (2015). NMR structure of the myristylated feline immunodeficiency virus matrix protein. Viruses.

[75] Watanabe S.M., Medina G.N., Eastep G.N., Ghanam R.H., Vlach J., Saad J.S., Carter C.A. (2018). The matrix domain of the Gag protein from avian sarcoma virus contains a PI(4,5)P2-binding site that targets Gag to the cell periphery. J. Biol. Chem..

[76] Ono A., Freed E.O. (2001). Plasma membrane rafts play a critical role in HIV-1 assembly and release. Proc. Natl. Acad. Sci. USA.

[77] Hogue I.B., Grover J.R., Soheilian F., Nagashima K., Ono A. (2011). Gag induces the coalescence of clustered lipid rafts and tetraspanin-enriched microdomains at HIV-1 assembly sites on the plasma membrane. J. Virol..

[78] Brügger B., Glass B., Haberkant P., Leibrecht I., Wieland F.T., Krausslich H.-G. (2006). The HIV lipidome: A raft with an unusual composition. Proc. Natl. Acad. Sci. USA.

[79] Charlier L., Louet M., Chaloin L., Fuchs P., Martinez J., Muriaux D., Favard C., Floquet N. (2014). Coarse-Grained Simulations of the HIV-1 Matrix Protein Anchoring: Revisiting Its Assembly on Membrane Domains. Biophys. J..

[80] Yandrapalli N., Lubart Q., Tanwar H.S., Picart C., Mak J., Muriaux D., Favard C. (2016). Self assembly of HIV-1 Gag protein on lipid membranes generates PI(4,5)P2/Cholesterol nanoclusters. Sci. Rep..

[81] Favard C., Chojnacki J., Merida P., Yandrapalli N., Mak J., Eggeling C., Muriaux D. (2019). HIV-1 Gag specifically restricts PI(4,5)P2 and cholesterol mobility in living cells creating a nanodomain platform for virus assembly. Sci. Adv..

[82] Alfadhli A., Still A., Barklis E. (2009). Analysis of Human Immunodeficiency Virus Type 1 Matrix Binding to Membranes and Nucleic Acids. J. Virol..

[83] Ehrlich L.S., Fong S., Scarlata S., Zybarth G., Carter C. (1996). Partitioning of HIV-1 Gag and Gag-related proteins to membranes. Biochemistry.

[84] Scarlata S., Ehrlich L.S., Carter C.A. (1998). Membrane-Induced Alterations in HIV-1 Gag and Matrix Protein-Protein Interactions. J. Mol. Biol..

[85] Mariani C., Desdouits M., Favard C., Benaroch P., Muriaux D.M. (2014). Role of Gag and lipids during HIV-1 assembly in CD4(+) T cells and macrophages. Front. Microbiol..

[86] Kutluay S.B., Zang T., Blanco-Melo D., Powell C., Jannain D., Errando M., Bieniasz P.D. (2014). Global Changes in the RNA Binding Specificity of HIV-1 Gag Regulate Virion Genesis. Cell.

[87] Inlora J., Collins D.R., Trubin M.E., Chung J.Y., Ono A. (2014). Membrane binding and subcellular localization of retroviral Gag proteins are differentially regulated by MA interactions with phosphatidylinositol-(4,5)-bisphosphate and RNA. mBio.

[88] Saad J.S., Loeliger E., Luncsford P., Liriano M., Tai J., Kim A., Miller J., Joshi A., Freed E.O., Summers M.F. (2007). Point mutations in the HIV-1 matrix protein turn off the myristyl switch. J. Mol. Biol..

[89] Vlach J., Saad J.S. (2013). Trio engagement via plasma membrane phospholipids and the myristoyl moiety governs HIV-1 matrix binding to bilayers. Proc. Natl. Acad. Sci. USA.

[90] Vlach J., Eastep G.N., Ghanam R.H., Watanabe S.M., Carter C.A., Saad J.S. (2018). Structural basis for targeting avian sarcoma virus Gag polyprotein to the plasma membrane for virus assembly. J. Biol. Chem..

[91] Mercredi P.Y., Bucca N., Loeliger B., Gaines C.R., Mehta M., Bhargava P., Tedbury P.R., Charlier L., Floquet N., Muriaux D. (2016). Structural and Molecular Determinants of Membrane Binding by the HIV-1 Matrix Protein. J. Mol. Biol..

[92] Anraku K., Fukuda R., Takamune N., Misumi S., Okamoto Y., Otsuka M., Fujita M. (2010). Highly sensitive analysis of the interaction between HIV-1 Gag and phosphoinositide derivatives based on surface plasmon resonance. Biochemistry.

[93] Shkriabai N., Datta S.A., Zhao Z., Hess S., Rein A., Kvaratskhelia M. (2006). Interactions of HIV-1 Gag with assembly cofactors. Biochemistry.

[94] Fernandes F., Chen K., Ehrlich L.S., Jin J., Chen M.H., Medina G.N., Symons M., Montelaro R., Donaldson J., Tjandra N. (2011). Phosphoinositides direct equine infectious anemia virus gag trafficking and release. Traffic.

[95] Murphy R.E., Samal A.B., Vlach J., Mas V., Prevelige P.E., Saad J.S. (2019). Structural and biophysical characterizations of HIV-1 matrix trimer binding to lipid nanodiscs shed light on virus assembly. J. Biol. Chem..

[96] Borch J., Hamann T. (2009). The nanodisc: A novel tool for membrane protein studies. Biol. Chem..

[97] Bayburt T.H., Sligar S.G. (2010). Membrane protein assembly into Nanodiscs. FEBS Lett..

[98] Hagn F., Etzkorn M., Raschle T., Wagner G. (2013). Optimized phospholipid bilayer nanodiscs facilitate high-resolution structure determination of membrane proteins. J. Am. Chem. Soc..

[99] Kobashigawa Y., Harada K., Yoshida N., Ogura K., Inagaki F. (2011). Phosphoinositide-incorporated lipid-protein nanodiscs: A tool for studying protein-lipid interactions. Anal. Biochem..

[100] Ritchie T.K., Grinkova Y.V., Bayburt T.H., Denisov I.G., Zolnerciks J.K., Atkins W.M., Sligar S.G. (2009). Chapter 11-Reconstitution of membrane proteins in phospholipid bilayer nanodiscs. Methods Enzymol..

[101] Yokogawa M., Kobashigawa Y., Yoshida N., Ogura K., Harada K., Inagaki F. (2012). NMR Analyses of the Interaction between the FYVE Domain of Early Endosome Antigen 1 (EEA1) and Phosphoinositide Embedded in a Lipid Bilayer. J. Biol. Chem..

[102] Ganser-Pornillos B.K., Cheng A., Yeager M. (2007). Structure of full-length HIV-1 CA: A model for the mature capsid lattice. Cell.

[103] Bharat T.A., Davey N.E., Ulbrich P., Riches J.D., de Marco A., Rumlova M., Sachse C., Ruml T., Briggs J.A. (2012). Structure of the immature retroviral capsid at 8 Å resolution by cryo-electron microscopy. Nature.

[104] Zhao G., Perilla J.R., Yufenyuy E.L., Meng X., Chen B., Ning J., Ahn J., Gronenborn A.M., Schulten K., Aiken C. (2013). Mature HIV-1 capsid structure by cryo-electron microscopy and all-atom molecular dynamics. Nature.

[105] Bharat T.A., Castillo Menendez L.R., Hagen W.J., Lux V., Igonet S., Schorb M., Schur F.K., Krausslich H.G., Briggs J.A. (2014). Cryo-electron microscopy of tubular arrays of HIV-1 Gag resolves structures essential for immature virus assembly. Proc. Natl. Acad. Sci. USA.

[106] Schur F.K., Hagen W.J., Rumlova M., Ruml T., Muller B., Krausslich H.G., Briggs J.A. (2015). Structure of the immature HIV-1 capsid in intact virus particles at 8.8 A resolution. Nature.

[107] Alfadhli A., Barklis R.L., Barklis E. (2009). HIV-1 matrix organizes as a hexamer of trimers on membranes containing phosphatidylinositol-(4,5)-bisphosphate. Virology.

[108] Tedbury P.R., Novikova M., Ablan S.D., Freed E.O. (2016). Biochemical evidence of a role for matrix trimerization in HIV-1 envelope glycoprotein incorporation. Proc. Natl. Acad. Sci. USA.

[109] Bouamr F., Scarlata S., Carter C.A. (2003). Role of myristylation in HIV-1 Gag assembly. Biochemistry.

[110] Alfadhli A., Huseby D., Kapit E., Colman D., Barklis E. (2007). Human Immunodeficiency Virus Type 1 Matrix Protein Assembles on Membranes as a Hexamer. J. Virol..

[111] Checkley M.A., Luttge B.G., Freed E.O. (2011). HIV-1 envelope glycoprotein biosynthesis, trafficking, and incorporation. J. Mol. Biol..

[112] Otteken A., Earl P.L., Moss B. (1996). Folding, assembly, and intracellular trafficking of the human immunodeficiency virus type 1 envelope glycoprotein analyzed with monoclonal antibodies recognizing maturational intermediates. J. Virol..

[113] Chen B. (2019). Molecular Mechanism of HIV-1 Entry. Trends Microbiol..

[114] Kirschman J., Qi M., Ding L., Hammonds J., Dienger-Stambaugh K., Wang J.J., Lapierre L.A., Goldenring J.R., Spearman P. (2018). HIV-1 Envelope Glycoprotein Trafficking through the Endosomal Recycling Compartment Is Required for Particle Incorporation. J. Virol..

[115] Qi M., Williams J.A., Chu H., Chen X., Wang J.J., Ding L., Akhirome E., Wen X., Lapierre L.A., Goldenring J.R. (2013). Rab11-FIP1C and Rab14 direct plasma membrane sorting and particle incorporation of the HIV-1 envelope glycoprotein complex. PLoS Pathog..

[116] Qi M., Chu H., Chen X., Choi J., Wen X., Hammonds J., Ding L., Hunter E., Spearman P. (2015). A tyrosine-based motif in the HIV-1 envelope glycoprotein tail mediates cell-type- and Rab11-FIP1C-dependent incorporation into virions. Proc. Natl. Acad. Sci. USA.

[117] Pancera M., Majeed S., Ban Y.E., Chen L., Huang C.C., Kong L., Kwon Y.D., Stuckey J., Zhou T., Robinson J.E. (2010). Structure of HIV-1 gp120 with gp41-interactive region reveals layered envelope architecture and basis of conformational mobility. Proc. Natl. Acad. Sci. USA.

[118] Merk A., Subramaniam S. (2013). HIV-1 envelope glycoprotein structure. Curr. Opin. Struct. Biol..

[119] Montero M., van Houten N.E., Wang X., Scott J.K. (2008). The membrane-proximal external region of the human immunodeficiency virus type 1 envelope: Dominant site of antibody neutralization and target for vaccine design. Microbiol. Mol. Biol. Rev..

[120] Alam S.M., Morelli M., Dennison S.M., Liao H.X., Zhang R., Xia S.M., Rits-Volloch S., Sun L., Harrison S.C., Haynes B.F. (2009). Role of HIV membrane in neutralization by two broadly neutralizing antibodies. Proc. Natl. Acad. Sci. USA.

[121] Pinto D., Fenwick C., Caillat C., Silacci C., Guseva S., Dehez F., Chipot C., Barbieri S., Minola A., Jarrossay D. (2019). Structural Basis for Broad HIV-1 Neutralization by the MPER-Specific Human Broadly Neutralizing Antibody LN01. Cell Host. Microbe.

[122] Fu Q., Shaik M.M., Cai Y., Ghantous F., Piai A., Peng H., Rits-Volloch S., Liu Z., Harrison S.C., Seaman M.S. (2018). Structure of the membrane proximal external region of HIV-1 envelope glycoprotein. Proc. Natl. Acad. Sci. USA.

[123] Blumenthal R., Durell S., Viard M. (2012). HIV entry and envelope glycoprotein-mediated fusion. J. Biol. Chem..

[124] Dev J., Park D., Fu Q., Chen J., Ha H.J., Ghantous F., Herrmann T., Chang W., Liu Z., Frey G. (2016). Structural basis for membrane anchoring of HIV-1 envelope spike. Science.

[125] Reichart T.M., Baksh M.M., Rhee J.K., Fiedler J.D., Sligar S.G., Finn M., Zwick M.B., Dawson P.E. (2016). Trimerization of the HIV transmembrane domain in lipid bilayers modulates broadly neutralizing antibody binding. Angew. Chem. Int. Edit..

[126] Dai Z., Tao Y., Liu N., Brenowitz M.D., Girvin M.E., Lai J.R. (2015). Conditional trimerization and lytic activity of HIV-1 gp41 variants containing the membrane-associated segments. Biochemistry.

[127] Lee J.H., Ozorowski G., Ward A.B. (2016). Cryo-EM structure of a native, fully glycosylated, cleaved HIV-1 envelope trimer. Science.

[128] Chiliveri S.C., Louis J.M., Ghirlando R., Baber J.L., Bax A. (2018). Tilted, Uninterrupted, Monomeric HIV-1 gp41 Transmembrane Helix from Residual Dipolar Couplings. J. Am. Chem. Soc..

[129] Postler T.S., Desrosiers R.C. (2013). The tale of the long tail: The cytoplasmic domain of HIV-1 gp41. J. Virol..

[130] Kirchhoff F., Kestler H., Desrosiers R.C. (1994). Upstream U3 sequences in simian immunodeficiency virus are selectively deleted in vivo in the absence of an intact nef gene. J. Virol..

[131] Kirchoff F., Greenough T.C., Brettler D.B., Sullivan J.L., Desrosiers R.C. (1995). Absence of intact nef sequences in a long-term servivor with nonprogressive HIV-1 infection. N. Engl. J. Med..

[132] Srinivas S.K., Srinivas R.V., Anantharamaiah G.M., Compans R.W., Segrest J.P. (1993). Cytosolic domain of the human immunodeficiency virus envelope glycoproteins binds to calmodulin and inhibits calmodulin-regulated proteins. J. Biol. Chem..

[133] Radding W., Pan Z.Q., Hunter E., Johnston P., Williams J.P., McDonald J.M. (1996). Expression of HIV-1 Envelope Glycoprotein Alters Cellular Calmodulin. Biochem. Biophys. Res. Commun..

[134] Wyss S., Berlioz-Torrent C., Boge M., Blot G., Höning S., Benarous R., Thali M. (2001). The highly conserved C-terminal dileucine motif in the cytosolic domain of the human immunodeficiency virus type 1 envelope glycoprotein is critical for its association with the AP-1 clathrin adapter. J. Virol..

[135] Ohno H., Aguilar R.C., Fournier M.C., Hennecke S., Cosson P., Bonifacino J.S. (1997). Interaction of endocytic signals from the HIV-1 envelope glycoprotein complex with members of the adaptor medium chain family. Virology.

[136] Boge M., Wyss S., Bonifacino J.S., Thali M. (1998). A membrane-proximal tyrosine-based signal mediates internalization of the HIV-1 envelope glycoprotein via interaction with the AP-2 clathrin adaptor. J. Biol. Chem..

[137] Berlioz-Torrent C., Shacklett B.L., Erdtmann L., Delamarre L., Bouchaert I., Sonigo P., Dokhelar M.C., Benarous R. (1999). Interactions of the cytoplasmic domains of human and simian retroviral transmembrane proteins with components of the clathrin adaptor complexes modulate intracellular and cell surface expression of envelope glycoproteins. J. Virol..

[138] Tedbury P.R., Ablan S.D., Freed E.O. (2013). Global Rescue of Defects in HIV-1 Envelope Glycoprotein Incorporation: Implications for Matrix Structure. PLoS Pathog..

[139] Tedbury P.R., Freed E.O. (2014). The role of matrix in HIV-1 envelope glycoprotein incorporation. Trends Microbiol..

[140] Tedbury P.R., Freed E.O. (2015). The cytoplasmic tail of retroviral envelope glycoproteins. Prog. Mol. Biol. Transl. Sci..

[141] Costin J.M., Rausch J.M., Garry R.F., Wimley W.C. (2007). Viroporin potential of the lentivirus lytic peptide (LLP) domains of the HIV-1 gp41 protein. Virol. J..

[142] Steckbeck J.D., Sun C., Sturgeon T.J., Montelaro R.C. (2010). Topology of the C-terminal tail of HIV-1 gp41: Differential exposure of the Kennedy epitope on cell and viral membranes. PLoS ONE.

[143] Boscia A.L., Akabori K., Benamram Z., Michel J.A., Jablin M.S., Steckbeck J.D., Montelaro R.C., Nagle J.F., Tristram-Nagle S. (2013). Membrane structure correlates to function of LLP2 on the cytoplasmic tail of HIV-1 gp41 protein. Biophys. J..

[144] Steckbeck J.D., Kuhlmann A.S., Montelaro R.C. (2013). C-terminal tail of human immunodeficiency virus gp41: Functionally rich and structurally enigmatic. J. Gen. Virol..

[145] Steckbeck J.D., Sun C., Sturgeon T.J., Montelaro R.C. (2013). Detailed topology mapping reveals substantial exposure of the "cytoplasmic" C-terminal tail (CTT) sequences in HIV-1 Env proteins at the cell surface. PLoS ONE.

[146] Miller M.A., Garry R.F., Jaynes J.M., Montelaro R.C. (1991). A structural correlation between lentivirus transmembrane proteins and natural cytolytic peptides. AIDS Res. Hum. Retroviruses.

[147] Srinivas S.K., Srinivas R.V., Anantharamaiah G.M., Segrest J.P., Compans R.W. (1992). Membrane interactions of synthetic peptides corresponding to amphipathic helical segments of the human immunodeficiency virus type-1 envelope glycoprotein. J. Biol. Chem..

[148] Steckbeck J.D., Craigo J.K., Barnes C.O., Montelaro R.C. (2011). Highly conserved structural properties of the C-terminal tail of HIV-1 gp41 protein despite substantial sequence variation among diverse clades: Implications for functions in viral replication. J. Biol. Chem..

[149] Viard M., Ablan S.D., Zhou M., Veenstra T.D., Freed E.O., Raviv Y., Blumenthal R. (2008). Photoinduced reactivity of the HIV-1 envelope glycoprotein with a membrane-embedded probe reveals insertion of portions of the HIV-1 Gp41 cytoplasmic tail into the viral membrane. Biochemistry.

[150] Bültmann A., Muranyi W., Seed B., Haas J. (2001). Identification of two sequences in the cytoplasmic tail of the human immunodeficiency virus type 1 envelope glycoprotein that inhibit cell surface expression. J. Virol..

[151] Kalia V., Sarkar S., Gupta P., Montelaro R.C. (2003). Rational site-directed mutations of the LLP-1 and LLP-2 lentivirus lytic peptide domains in the intracytoplasmic tail of human immunodeficiency virus type 1 gp41 indicate common functions in cell-cell fusion but distinct roles in virion envelope incorporation. J. Virol..

[152] Lee S.F., Ko C.Y., Wang C.T., Chen S.S. (2002). Effect of point mutations in the N terminus of the lentivirus lytic peptide-1 sequence of human immunodeficiency virus type 1 transmembrane protein gp41 on Env stability. J. Biol. Chem..

[153] Piller S.C., Dubay J.W., Derdeyn C.A., Hunter E. (2000). Mutational analysis of conserved domains within the cytoplasmic tail of gp41 from human immunodeficiency virus type 1: Effects on glycoprotein incorporation and infectivity. J. Virol..

[154] Murphy R.E., Samal A.B., Vlach J., Saad J.S. (2017). Solution Structure and Membrane Interaction of the Cytoplasmic Tail of HIV-1 gp41 Protein. Structure.

[155] Cleveland S.M., McLain L., Cheung L., Jones T.D., Hollier M., Dimmock N.J. (2003). A region of the C-terminal tail of the gp41 envelope glycoprotein of human immunodeficiency virus type 1 contains a neutralizing epitope: Evidence for its exposure on the surface of the virion. J. Gen. Virol..

[156] Hollier M.J., Dimmock N.J. (2005). The C-terminal tail of the gp41 transmembrane envelope glycoprotein of HIV-1 clades A, B, C, and D may exist in two conformations: An analysis of sequence, structure, and function. Virology.

[157] Kuhlmann A.S., Steckbeck J.D., Sturgeon T.J., Craigo J.K., Montelaro R.C. (2014). Unique functional properties of conserved arginine residues in the lentivirus lytic peptide domains of the C-terminal tail of HIV-1 gp41. J. Biol. Chem..

[158] Akari H., Fukumori T., Adachi A. (2000). Cell-dependent requirement of human immunodeficiency virus type 1 gp41 cytoplasmic tail for Env incorporation into virions. J. Virol..

[159] Murakami T., Freed E.O. (2000). The long cytoplasmic tail of gp41 is required in a cell type-dependent manner for HIV-1 envelope glycoprotein incorporation into virions. Proc. Natl. Acad. Sci. USA.

[160] Hales C.M., Griner R., Hobdy-Henderson K.C., Dorn M.C., Hardy D., Kumar R., Navarre J., Chan E.K., Lapierre L.A., Goldenring J.R. (2001). Identification and characterization of a family of Rab11-interacting proteins. J. Biol. Chem..

[161] Roy N.H., Chan J., Lambele M., Thali M. (2013). Clustering and mobility of HIV-1 Env at viral assembly sites predict its propensity to induce cell-cell fusion. J. Virol..

[162] Muranyi W., Malkusch S., Müller B., Heilemann M., Kräusslich H.G. (2013). Super-Resolution Microscopy Reveals Specific Recruitment of HIV-1 Envelope Proteins to Viral Assembly Sites Dependent on the Envelope C-Terminal Tail. PLoS Pathog..

[163] Buttler C.A., Pezeshkian N., Fernandez M.V., Aaron J., Norman S., Freed E.O., van Engelenburg S.B. (2018). Single molecule fate of HIV-1 envelope reveals late-stage viral lattice incorporation. Nat. Commun..

[164] Owens R.J., Compans R.W. (1989). Expression of the human immunodeficiency virus envelope glycoprotein is restricted to basolateral surfaces of polarized epithelial cells. J. Virol..

[165] Owens R.J., Dubay J.W., Hunter E., Compans R.W. (1991). Human Immunodeficiency Virus Envelope Protein Determines the Site of Virus Release in Polarized Epithelial Cells. Proc. Natl. Acad. Sci. USA.

[166] Lodge R., Gottlinger H., Gabuzda D., Cohen E.A., Lemay G. (1994). The intracytoplasmic domain of gp41 mediates polarized budding of human immunodeficiency virus type 1 in MDCK cells. J. Virol..

[167] Kiernan R.E., Freed E.O. (1998). Cleavage of the murine leukemia virus transmembrane env protein by human immunodeficiency virus type 1 protease: Transdominant inhibition by matrix mutations. J. Virol..

[168] Song Y.E., Olinger G.Y., Janaka S.K., Johnson M.C. (2019). Sequence Determinants in Gammaretroviral Env Cytoplasmic Tails Dictate Virus-Specific Pseudotyping Compatibility. J. Virol..

[169] Yu X., Yuan X., Matsuda Z., Lee T.-H., Essex M. (1992). The Matrix Protein of Human Immunodeficiency Virus Type I is Required for Incorporation of Viral Envelope Protein into Mature Virions. J. Virol..

[170] Freed O.E., Martin A.M. (1995). Virion Incorporation of Envelope Glycoproteins with Long but Not Short Cytoplasmic Tails Is Blocked by Specific, Single Amino Acid Substitutions in the Human Immunodeficiency Virus Type 1 Matrix. J. Virol..

[171] Dorfman T., Mammano F., Haseltine W.A., Göttlinger H.G. (1994). Role of the Matrix Protein in the Virion Association of the Human Immunodeficiency Virus Type 1 Envelope Glycoprotein. J. Virol..

[172] Freed E.O., Martin A.M. (1996). Domains of the Human Immonodeficiency Virus Type 1 Matrix and gp41 Cytoplasmic Tail Required for Envelope Incorporation into Virions. J. Virol..

[173] Cosson P. (1996). Direct interaction between the envelope and matrix proteins of HIV-1. EMBO J..

[174] Ono A., Huang M., Freed E.O. (1997). Characterization of human immunodeficiency virus type 1 matrix revertants: Effects on virus assembly, Gag processing, and Env incorporation into virions. J. Virol..

[175] Murakami T., Ablan S., Freed E.O., Tanaka Y. (2004). Regulation of human immunodeficiency virus type 1 Env-mediated membrane fusion by viral protease activity. J. Virol..

[176] Wyma D.J., Jiang J., Shi J., Zhou J., Lineberger J.E., Miller M.D., Aiken C. (2004). Coupling of human immunodeficiency virus type 1 fusion to virion maturation: A novel role of the gp41 cytoplasmic tail. J. Virol..

[177] Pezeshkian N., Groves N.S., van Engelenburg S.B. (2019). Single-molecule imaging of HIV-1 envelope glycoprotein dynamics and Gag lattice association exposes determinants responsible for virus incorporation. Proc. Natl. Acad. Sci. USA.

[178] Alfadhli A., Staubus A.O., Tedbury P.R., Novikova M., Freed E.O., Barklis E. (2019). Analysis of HIV-1 Matrix-Envelope Cytoplasmic Tail Interactions. J. Virol..

[179] Tedbury P.R., Novikova M., Alfadhli A., Hikichi Y., Kagiampakis I., KewalRamani V.N., Barklis E., Freed E.O. (2019). HIV-1 Matrix Trimerization-Impaired Mutants Are Rescued by Matrix Substitutions That Enhance Envelope Glycoprotein Incorporation. J. Virol..

[180] Tedbury P.R., Mercredi P.Y., Gaines C.R., Summers M.F., Freed E.O. (2015). Elucidating the mechanism by which compensatory mutations rescue an HIV-1 matrix mutant defective for gag membrane targeting and envelope glycoprotein incorporation. J. Mol. Biol..

[181] Alfadhli A., Mack A., Ritchie C., Cylinder I., Harper L., Tedbury P.R., Freed E.O., Barklis E. (2016). Trimer Enhancement Mutation Effects on HIV-1 Matrix Protein Binding Activities. J. Virol..

[182] Waheed A.A., Freed E.O. (2012). HIV Type 1 Gag as a Target for Antiviral Therapy. AIDS Res. Hum. Retroviruses.

[183] Dick A., Cocklin S. (2020). Recent Advances in HIV-1 Gag Inhibitor Design and Development. Molecules.

[184] LaPlante S.R., Forgione P., Boucher C., Coulombe R., Gillard J., Hucke O., Jakalian A., Joly M.A., Kukolj G., Lemke C. (2014). Enantiomeric atropisomers inhibit HCV polymerase and/or HIV matrix: Characterizing hindered bond rotations and target selectivity. J. Med. Chem..

[185] Zentner I., Sierra L.J., Fraser A.K., Maciunas L., Mankowski M.K., Vinnik A., Fedichev P., Ptak R.G., Martín-García J., Cocklin S. (2013). Identification of a Small-Molecule Inhibitor of HIV-1 Assembly that Targets the Phosphatidylinositol (4,5)-bisphosphate Binding Site of the HIV-1 Matrix Protein. ChemMedChem.

[186] Zentner I., Sierra L.J., Maciunas L., Vinnik A., Fedichev P., Mankowski M.K., Ptak R.G., Martín-García J., Cocklin S. (2013). Discovery of a small-molecule antiviral targeting the HIV-1 matrix protein. Bioorg. Med. Chem. Lett..

